# Sonographic Signs of Adenomyosis Are Prevalent in Women Undergoing Surgery for Endometriosis and May Suggest a Higher Risk of Infertility

**DOI:** 10.1155/2017/8967803

**Published:** 2017-09-18

**Authors:** Vered H. Eisenberg, Nissim Arbib, Eyal Schiff, Motti Goldenberg, Daniel S. Seidman, David Soriano

**Affiliations:** ^1^Department of Obstetrics and Gynecology, Sheba Medical Center, Tel Hashomer, Israel; ^2^Sackler Faculty of Medicine, Tel Aviv University, Tel Aviv, Israel

## Abstract

**Objectives:**

To determine the prevalence of ultrasound features suggestive of adenomyosis in women undergoing surgery for endometriosis compared with a control group of healthy women without endometriosis.

**Methods:**

Retrospective case-control study comparing women with intractable pain or infertility, who underwent transvaginal ultrasound and subsequent laparoscopic surgery, with a control group of healthy women without a previous history of endometriosis. A diagnosis of adenomyosis on TVUS was made based on asymmetrical myometrial thickening, linear striations, myometrial cysts, hyperechoic islands, irregular endometrial-myometrial junction, parallel shadowing, and localized adenomyomas and analyzed for one sign and for three or more signs.

**Results:**

The study and control groups included 94 and 60 women, respectively. In the study group, women were younger and had more dysmenorrhea and infertility symptoms. The presence of any sonographic feature of adenomyosis, as well as three or more signs, was found to be more prevalent in the study group, which persisted after controlling for age, for all features but linear striations. Women in the study group who had five or more sonographic features of adenomyosis had more than a threefold risk of suffering from infertility (OR = 3.19, *p* = 0.015, 95% CI; 1.25–8.17). There was no association with disease severity at surgery.

**Conclusions:**

Sonographic features of adenomyosis are more prevalent in women undergoing surgery for endometriosis compared to healthy controls. Women with more than five features had an increased risk of infertility.

## 1. Introduction

Adenomyosis is a benign disorder of the uterus that is defined as the presence of endometrial glands and stroma within the uterine myometrium. Reports of the prevalence of adenomyosis are highly heterogenous and inconsistent and are dependent on the population studied and the methodology used for evaluation. Many studies rely on histological findings in women undergoing hysterectomies and report a higher prevalence, as hysterectomies are performed on women with a known indication [1–4]. Adenomyosis is most often found in women between 40 and 50 years of age [1]. This age range may be explained by the more common performance of hysterectomies in this age group, but may also be attributed to prolonged life-time exposure to hormones [5]. The most commonly reported associated symptoms are abnormal uterine bleeding and dysmenorrhea that occur in approximately 65% of patients [5]. Adenomyosis often coexists with deep endometriosis. The association between adenomyosis, endometriosis, and infertility is still under debate and the mechanism is poorly understood. Patients with coexisting deep infiltrating endometriosis and uterine adenomyosis may constitute a subgroup with a particularly poor reproductive prognosis [6–10]. A recent meta-analysis described a 68% reduction in the likelihood of pregnancy in women seeking conception after surgery for rectovaginal and colorectal endometriosis [11].

The improved resolution of transvaginal ultrasound (TVUS) probes enables a detailed and thorough assessment of the uterine structure with detection of features, which have not been previously seen. Recent studies report the prevalence of adenomyosis based on the imaging method that was used, such as TVUS [12–14] or magnetic resonance imaging (MRI) [15–17]. Adenomyosis may be reliably detected by both TVUS and MRI without the need for histological examination of a biopsy specimen [15, 18]. The advantages of TVUS over MRI are its wide availability and economy. Recent studies advocate that TVUS be used as the first line imaging modality in women undergoing preoperative evaluation before endometriosis surgery, for determining the extent and severity of the disease, and mapping the way for the surgeon [19–21]. TVUS is considered an accurate diagnostic tool for the diagnosis of adenomyosis, and can therefore be used as standard clinical practice for the noninvasive diagnosis of adenomyosis [12, 18, 22–24]. The most commonly described two dimensional (2D) TVUS findings for adenomyosis are a heterogenous myometrium, abnormal myometrial echo texture, myometrial cysts, a globular and/or asymmetric uterus, ill-defined margins between the endometrium and the myometrium, echogenic linear striations, and focal adenomyomas [12–14, 25]. Three-dimensional (3D) TVUS also allows clear visualization of the endometrial-myometrial junctional (EMJ) zone and enables early diagnosis of adenomyosis [26, 27].

While the prevalence of adenomyosis in women undergoing surgery has been described, there is less data on the association with endometriosis and on the prevalence in asymptomatic women. The aim of our study was to determine the prevalence of ultrasound features suggestive of adenomyosis in women undergoing laparoscopic surgery for endometriosis in a tertiary referral center compared with a control group of healthy women without endometriosis attending a medical screening facility, using 2D and 3D TVUS. Our secondary aim was to explore the relationship between these sonographic features with demographic parameters and symptoms, particularly infertility.

## 2. Patients and Methods

### 2.1. Patients and Setting

We retrospectively studied women who were referred to our endometriosis center between November 2011 and March 2013 and underwent a dedicated TVUS and subsequent laparoscopic surgery. Out of the 250 patients who were examined during the study period, 94 underwent surgery at our institution and were included in the analysis. The indication for surgery was either intractable pain not responsive to conservative management or persistent infertility. The remaining women either did not qualify for surgery, preferred conservative treatment, or were operated on at another institution and therefore were not included in the analysis. The patients' demographic information, clinical history, and symptoms were obtained from the electronic hospital records and from outpatient referral documents and included: age, body mass index (BMI), parity, previous cesarean sections, previous surgery for endometriosis, smoking history, dysmenorrhea, dyspareunia, urinary and gastrointestinal symptoms, infertility history, previous fertility treatment and type, and number of previous in vitro fertilization (IVF) cycles. The control group consisted of reproductive age women attending a general medical screening facility in our institution, who underwent a TVUS as part of the annual checkup, on the days that the expert sonographer performed the clinical round. Women were included at random without preselection. Most of the women attending the medical screening facility were past their reproductive period, so it was difficult to find eligible patients. Women with a previous history of endometriosis, previous surgery for endometriosis or following a hysterectomy, were excluded from the control group analysis.

Ethical approval was obtained from our local research ethics committee (IRB). Written informed consent was not required as the ultrasound assessment was offered as part of standard clinical care at our center and in the medical screening facility. No procedure was performed for the purpose of the study and no identifying information is included in the data presented here.

### 2.2. Evaluation of Adenomyosis and Endometriosis

A TVUS scan was carried out using a 7.5 MHz probe with 2D/3D capabilities (Voluson 730 and E6, and P6, GE Medical Systems, Villach, Austria), in a standardized way by the same imaging expert. The examination included a thorough evaluation of all pelvic viscera and was performed at any time of the menstrual cycle regardless of hormonal therapy. Bowel preparation was not utilized. The uterus was studied in a mid-sagittal plane identifying the uterine cavity and cervical canal, moving to the right and left in order to cover the entire uterine cavity. The probe was then rotated 90 degrees to the left to view the uterus in the transverse plane. The myometrium was thoroughly evaluated for any abnormalities in all planes. The analysis for both groups was based on stored 2D images and cine loops. All women were examined by the same expert sonographer using the same methodology. We did have 3D capabilities available for the study group but decided not to use them for the sake of equal comparison using the same modalities for both groups.

**Figure 1:**
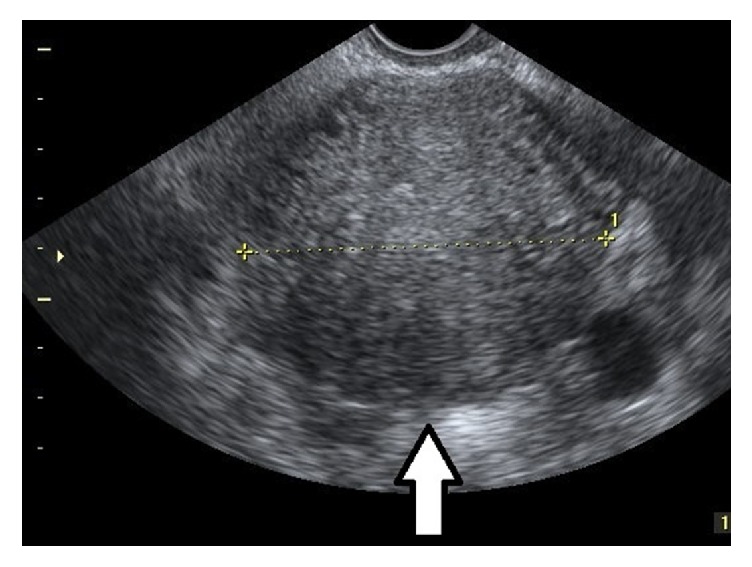
Parallel shadowing. 2D image in transverse view of a uterus with parallel hypoechoic lines through the myometrium (arrow).

**Figure 2:**
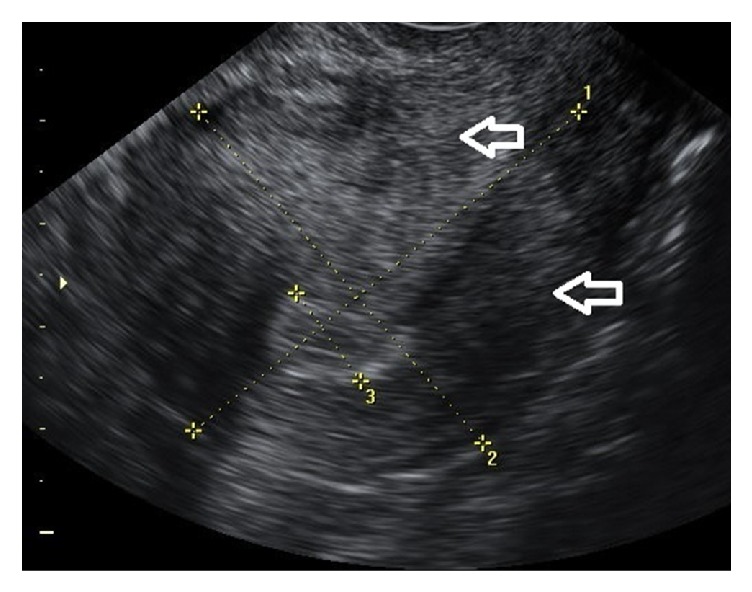
Asymmetrical myometrial thickening. 2D longitudinal view of a uterus with asymmetrical distances from the endometrium to the anterior and posterior serosal surfaces (arrows).

**Figure 3:**
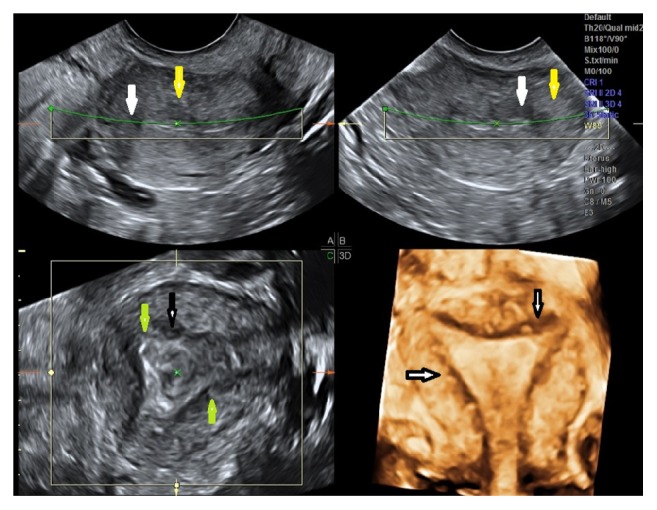
Severe adenomyosis with multiple sonographic signs: multiplanar and 3D rendering of an anteverted uterus with multiple sonographic signs: myometrial cysts (white arrow), hyperechoic islands (yellow arrow), linear striations (green arrow), and irregular EMJ (black arrow).

**Figure 4:**
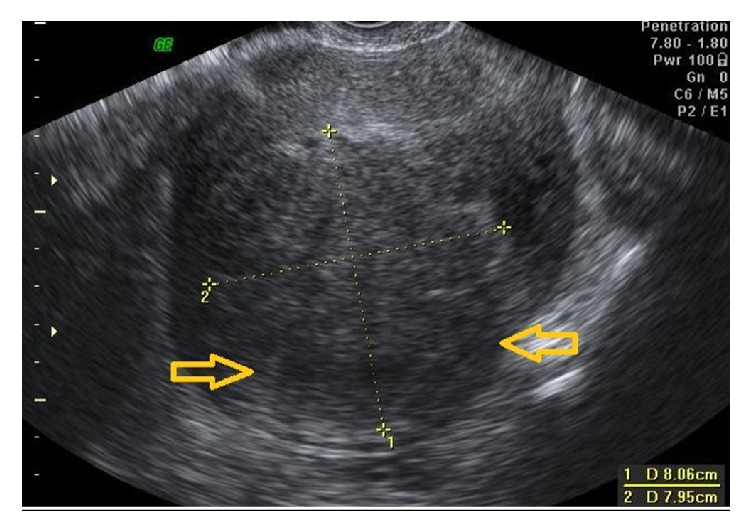
Localized adenomyoma. 2D image of an anteverted uterus with a localized adenomyoma in the posterior fundal wall (between yellow arrows).

A diagnosis of adenomyosis was made at the time of the exam, when any one of the following features was present: asymmetrical myometrial thickening (in the absence of fibroids), parallel shadowing, myometrial cysts, hyperechoic islands, irregular endometrial-myometrial junction (EMJ), linear striations, and localized adenomyomas (Figures 1–4). An adenomyoma was defined as a nodular, heterogeneous myometrial mass with ill-defined borders [23]. These features were chosen because they are all recognized as reliable morphological sonographic markers for adenomyosis [12–14] and can be differentially diagnosed from fibroids [23, 28]. The accuracy of these findings was evaluated against the pathological report when available. In order to increase accuracy, we looked at a combination of features and calculated the same parameters for three or above and for five or above sonographic features.

A diagnosis of endometriosis on ultrasound was based on the presence of ovarian endometriomas, deeply infiltrative endometriotic nodules, signs of pelvic adhesions (kissing ovaries or absent sliding of viscera), or overt tubal disease [19–21, 27]. The severity of endometriosis at surgery was evaluated based on the Revised American Society for Reproductive Medicine (ASRM) Classification [29], and the histopathological reports were reviewed. We included only the women for whom we had histological confirmation of endometriosis.

### 2.3. Statistical Analysis

Statistical analysis was performed using SPSS software version 20 (SPSS Inc., IBM corporation, Chicago, IL, USA). Continuous variables were expressed as means ± SD or medians, while categorical variables were expressed as percentages. The Fisher exact test was used to detect differences in percentages and the Student* t*-test was used to compare means. Sensitivity, specificity, positive predictive value (PPV), negative predictive value (NPV), and accuracy were calculated for the diagnosis of adenomyosis on ultrasound. Associations between various demographic, symptomatic and clinical variables and disease severity at surgery, and the presence of adenomyosis on ultrasound were assessed using logistic regression, and univariate and multivariate analyses were performed. The analysis was performed for at least one sign, three signs or more, and five or above signs. The associations between sonographic features of adenomyosis and demographic variables were assessed using logistic regression for 3 models: without adjustment for variables, with adjustment for age, and with adjustment for age, smoking, BMI, and previous cesarean sections. Statistical significance was set at *p* < 0.05.

## 3. Results

### 3.1. Demographic and Clinical Characteristics

Ninety-four women were included in the study group, all of which underwent TVUS and subsequent laparoscopic surgery over the study period, and sixty women in the control group. Demographic data and patient symptoms are presented in Table 1. None of the women were menopausal. In the study group symptoms and complaints included dysmenorrhea (92.5%), dyspareunia (64.1%), urinary complaints (28.6%), gastrointestinal complaints (53.8%), and infertility (*37.2*%). All patients described long-standing symptoms before being referred to our center. Of the 94 women, 49 (52%) had undergone previous surgery for endometriosis. Twenty-five women (26.6%) had undergone IVF treatments prior to surgery, the median number of IVF treatments was 5 (range 1–16), with 17 women undergoing 3 cycles or more, without success. The indication for infertility treatment in all of these women was female infertility, and there were no cases of male factor infertility.

In the control group (see Table 1), women were older and more parous with a higher mean parity. None of the women were menopausal. Two women had undergone previous laparoscopy for indications other than endometriosis. There was less infertility and less need for IVF treatments, and only four women had undergone three IVF cycles or more.

### 3.2. Surgeries

All of the patients underwent laparoscopic surgery by a multidisciplinary team of trained endoscopic surgeons, which included urological and colorectal surgeons as required. The indication for surgery was intractable pain not amenable to conservative treatment or infertility. Adenomyosis or the concurrent presence of fibroids were not sole indications but could be complementary and thus did not affect surgical indication in itself. Fifty-seven (60.6%) of the women had endometriomas, 11 (11.7%) bladder nodules, 39 (41.5%) vaginal nodules, 48 (51.1%) pouch of Douglas obliteration, 20 (21.3%) bowel nodules (rectum, bowel, and pouch of Douglas), and 50 (53.2%) uterosacral ligament involvement. The mean disease severity (ASRM) score at surgery was 51.28 ± 38.25 (range 1–148), and the median ASRM stage was 4 (range 1–4). Fifteen (16%) patients had stage I, 4 (4.3%) stage II, 19 (20.2%) stage III, and 56 (59.6%) stage IV disease. Women with stage I or II disease also underwent surgery for intractable pain unresponsive to conservative management or infertility. Hysterectomies were performed only in 14 women who suffered from severe symptomatic adenomyosis and endometriosis who had completed family planning. Thus, histological confirmation of adenomyosis in hysterectomy specimens was available only in these women (15%), providing a sensitivity of 100%, specificity of 25%, positive predictive value of 89.5%, and negative predictive value of 100% for TVUS diagnosis of adenomyosis. Endometriosis was histologically confirmed in all of the women who were included in the analysis as mentioned above.

There were no surgeries in the control group.

### 3.3. Sonographic Features Suggestive of Adenomyosis

The prevalence of sonographic features suggestive of adenomyosis in the study and control groups is presented in Table 2. There was a high overall prevalence (89.4%) of sonographic signs of adenomyosis in women undergoing laparoscopic surgery for endometriosis, much higher than in controls. The presence of any sonographic feature of adenomyosis was found to be more prevalent in the group of women with endometriosis as compared to the control group, despite their younger age. Features that were significantly more prevalent in women undergoing surgery compared to the control group were parallel shadowing linear striations, irregularity of the EMJ, and focal adenomyomas. The prevalence of any sonographic sign of adenomyosis was found to increase with age in both groups (*p* < 0.01). The presence of three or above and of five or above sonographic features was found to be more prevalent in the group of women with endometriosis as compared to the control group, and both were statistically significant (*p* < 0.01).

### 3.4. Association between Sonographic Features of Adenomyosis and Demographic Variables

The associations between sonographic features of adenomyosis and demographic variables using logistic regression for the three chosen models (without adjustment for variables, with adjustment for age, and with adjustment for age, smoking, BMI, and previous cesarean sections) are presented in Table 3. For all features but linear striations, the OR of having a specific feature was higher in women undergoing surgery as compared to the control group. The most significant association was found for irregularity of the EMJ, and focal adenomyomas, followed by parallel shadowing. After adjusting for age, all associations became markedly stronger.

In the study group, we could not find a significant association between the number of sonographic signs and the presence of clinical symptoms (Pearson Correlation not significant). In an attempt to determine the severity of adenomyosis based on ultrasound findings, we stratified the number of adenomyosis signs into 5 signs and above compared to fewer sonographic signs and performed the logistic regression again (see Table 4). Women with 5 or more features suggestive of adenomyosis had more than a 3-fold risk of suffering from infertility (OR = 3.19, *p* = 0.015, 95% CI; 1.25–8.17), a highly significant association. A similar finding was observed for women with 3 or more features suggestive of adenomyosis (OR = 2.51, *p* = 0.007, 95% CI; 1.28–4.9). However, there was no significant relationship with endometriosis severity at surgery. Of the women in the study group, 82.5% had normal patent tubes on both sides during surgery, eliminating mechanical infertility as a main cause.

## 4. Discussion

In this study, we found a very high overall prevalence (89.4%) of sonographic signs of adenomyosis in women undergoing laparoscopic surgery for endometriosis, much higher than in controls. We further found it to increase with age in both groups. The features that were found to be more significant were an irregular EMJ and focal adenomyomas, followed by parallel shadowing. An important finding was that women with more than 5 signs indicative of adenomyosis had a 3-fold increased risk of suffering from infertility, independent of the surgical severity of endometriosis.

Another interesting finding was that adenomyosis was reasonably prevalent in the controls as well, which may be attributed to the older age of the control group, as adenomyosis is known to be more prevalent in women in their late reproductive period. Despite this disparity in age between the study and control groups, adenomyosis was still found to be more common in the study group. In order to overcome this surprising finding, we reevaluated our data against 3 features or more and against 5 features or more. And indeed, in the study group, there was a greater prevalence than in the control group, in accordance with our prestudy expectations. In the control group, the features that were found to be the most prevalent were myometrial cysts and hyperechoic islands. It is plausible that these are early features of adenomyosis or a result of continued hormonal exposure as women age, whereas other features may be a marker of more advanced disease or of the association with endometriosis. These observations merit further study.

Previous studies addressing the prevalence of adenomyosis were performed on a surgical cohort with histological confirmation following hysterectomy. Until recently, MRI was generally considered to be the gold-standard imaging modality for diagnosis of adenomyosis. However, recent studies involving TVUS imaging showed higher accuracy and comparable detection rates [18, 24, 25, 28, 30, 31]. More recent studies [14, 22] have highlighted the coexistence of adenomyosis and deep infiltrating endometriosis in approximately 40–50% of women; the latter study also showed that related symptoms persisted after surgery when adenomyosis was present. Several studies have previously confirmed an association between adenomyosis and endometriosis; thus, this is not unexpected [14, 24, 32]. Hysterectomies are rarely performed for pain in modern clinical practice, mainly because most women seeking therapy are young and desirous of fertility and are operated on for indications of intractable severe pain or infertility issues. For this reason, histological confirmation of imaging findings is not always possible. Ultrasound and MRI are at present the only noninvasive modalities for preoperative diagnosis of adenomyosis. Ultrasound is more accessible, cheaper, and not inferior to MRI, leading to the opinion that TVUS should be the primary tool for the noninvasive diagnosis of adenomyosis and that surgical confirmation is not mandatory, particularly in women desirous of fertility [5, 12, 14].

The hypothesis that adenomyosis and infertility may be linked is gaining wider acceptance as increasing evidence to this effect is produced [6, 26, 27, 33–35]. A recent meta-analysis evaluated IVF outcomes in women with adenomyosis and found a 68% reduction in the likelihood of clinical pregnancy at in vitro fertilization/intracytoplasmic sperm injection (IVF/ICSI), and more than double the risk of miscarriage in these women [34]. The detrimental effect of adenomyosis on IVF/ICSI outcome seems to be both in reduced pregnancy rates and in increased early pregnancy loss. Indeed, in our study there was a high rate of IVF treatments before surgery in the study group, which could have also been related to the presence of adenomyosis. Age may also have been a detrimental factor, although in general, the women were young. Specific treatment modalities aimed at alleviating adenomyosis and endometriosis that conserve uterine and ovarian function are important considerations in women desiring fertility. Accurate preoperative assessment of adenomyosis in women with endometriosis scheduled for surgery is imperative in order to preserve and plan reproductive management. Screening for adenomyosis before embarking on medically assisted reproductive procedures should be encouraged in this group with a high risk for infertility. Conversely fertility sparing surgical methods should be implemented in general and particularly if adenomyosis is found in a woman operated on for endometriosis.

The strength of our study is in the fact that a single operator specifically dedicated to endometriosis evaluation performed all of the TVUS examinations using a high frequency transvaginal probe and utilizing known diagnostic criteria, for both the study and the control group. The morphological diagnostic features that we used have been previously described as valid criteria for noninvasive diagnosis of adenomyosis [13–17]. These features were recently described in a statement paper by the Myometrial Pathology Using Ultrasonography Consensus Group (MUSA) [30]. A further strength of this study is our control group of healthy women, from which women who may have had symptoms suggestive of endometriosis were excluded. Additionally, we performed the evaluation for multiple features of adenomyosis in order to increase accuracy and overcome potential bias, particularly the one arising from the age discrepancy discussed above.

A weakness of this study is the retrospective design and the limited availability of histological confirmation, as these were mostly young women seeking fertility which influenced the low hysterectomy rates. However, the correlation between ultrasound diagnosis of adenomyosis and histological diagnosis in the women who did undergo hysterectomies was good and strongly supports the known validity of this modality for preoperative diagnosis, even though the number of hysterectomies was small. As stated previously, the recent consensus [30] implies that ultrasound can be a definitive diagnosis without the need for histological confirmation. Furthermore, the comparison with the control group addresses this caveat. There may be a slight selection bias in our study. The prevalence that was found is high, most probably because this is a highly selected population of women with severe endometriosis and severe symptoms that were selected for surgical treatment and for whom conservative management was not an option.

In conclusion, sonographic features of adenomyosis are highly prevalent in women undergoing surgery for endometriosis. A large number of sonographic signs of adenomyosis were found to be associated with a higher risk of suffering from infertility, regardless of endometriosis severity. This can be taken to mean that endometriosis severity is not the only predictor of fertility in these women. Furthermore, this may bear direct implications on tailoring patient-specific treatments, both before and after the operation, such as secondary prevention by hormonal therapy or choice and timing of fertility treatments. It would be interesting to conduct a prospective study utilizing this potential scoring system for adenomyosis in symptomatic and asymptomatic women, in order to confirm these findings. Further study may be indicated in order to evaluate the relationship between age and sonographic features of adenomyosis in a healthy control group, and to compare women with endometriosis undergoing surgery to those who do not. We plan to investigate these issues in future research.

## Figures and Tables

**Table 1:** Demographic data and symptoms in women undergoing surgery for endometriosis versus the control group.

	Endometriosis (*N* = 94)	Control (*N* = 60)	*p*
Age, mean ± SD, years	34.1 ± 6.0	42.7 ± 3.2	<0.001^*∗*^
BMI, mean ± SD, kg/m^2^	23.6 ± 4.8	23.8 ± 4.1	0.830
Parous (%)	42 (44.7%)	58 (96.7%)	<0.001^*∗*^
Parity, mean ± SD	0.9 ± 1.2	2.4 ± 0.9	<0.001^*∗*^
Previous cesarean section (%)	0.2 ± 0.6	0.3 ± 0.7	0.183
Smoker (%)	28 (29.8)	9 (15.0)	0.052
Previous laparoscopy (%)	47 (50.0)	2 (3.3)	<0.001^*∗*^
Dysmenorrhea (%)	86 (92.5)	14 (25.0)	<0.001^*∗*^
Infertility (%)	32 (35.6)	14 (23.3)	0.148
Previous IVF treatment (%)	25 (30%)	9 (15%)	0.038^*∗*^
Number of IVF cycles, mean ± SD	1.8 ± 3.9	0.6 ± 2.2	0.022^*∗*^

SD: standard deviation; IVF: in vitro fertilization treatment. ^*∗*^Statistically significant finding.

**Table 2:** Transvaginal ultrasound features suggestive of adenomyosis and their prevalence in women undergoing surgery for endometriosis versus controls.

	Endometriosis (*N* = 94)	Control (*N* = 60)	*p*
Asymmetrical myometrial thickening (%)	64 (68.1)	38 (63.3)	0.602
Myometrial cysts (%)	80 (85.1)	47 (78.3)	0.287
Parallel shadowing (%)	54 (57.5)	22 (36.7)	0.014^*∗*^
Hyperechoic islands (%)	76 (80.9)	46 (76.7)	0.547
Linear striations (%)	25 (26.6)	27 (45.0)	0.023^*∗*^
Irregular EMJ (%)	81 (86.2)	26 (43.3)	<0.001^*∗*^
Focal adenomyomas (%)	36 (38.3)	7 (11.7)	<0.001^*∗*^
Number of features, mean ± SD	4.4 ± 2.0	3.5 ± 2.3	0.009^*∗*^
Any feature (%)	84 (89.4)	47 (78.3)	0.068
Number of features ≥ 3 (%)	82 (87.2)	41 (68.3)	0.004^*∗*^
Number of features ≥ 5 (%)	54 (57.4)	21 (35)	0.007^*∗*^

EMJ: endometrial-myometrial junction; SD: standard deviation. ^*∗*^Statistically significant finding.

**Table 3:** The odds ratios for the association between sonographic features of adenomyosis in the study group versus the control group and demographic variables using logistic regression for the three chosen models: Model 1: unadjusted, without adjustment for variables; Model 2: adjusted for age; Model 3: adjusted for age, smoking, BMI, and previous cesarean sections.

	Model 1 (Unadjusted)				Model 2 (Adjusted for age)				Model 3 (Adjusted for age, smoking, BMI and CS)			
	OR	95% CI for OR		*p*	OR	95% CI for OR		*p*	OR	95% CI for OR		*p*
		LL	UL			LL	UL			LL	UL	
Any one feature	2.32	0.95	5.70	0.066	9.02	2.04	39.95	0.004^*∗*^	13.01	2.46	68.78	0.003^*∗*^
Asymmetrical thickening	1.23	0.62	2.44	0.543	1.98	0.79	4.98	0.144	2.20	0.85	5.71	0.105
Myometrial cysts	1.58	0.68	3.65	0.283	3.05	0.92	10.16	0.069	4.02	1.13	14.35	0.032^*∗*^
Parallel shadowing	2.33	1.19	4.54	0.013^*∗*^	5.84	2.29	14.91	<0.001^*∗*^	6.28	2.37	16.65	<0.001^*∗*^
Hyperechoic islands	1.28	0.58	2.83	0.533	2.28	0.76	6.86	0.141	2.47	0.77	7.86	0.127
Linear striations	0.44	0.22	0.88	0.020^*∗*^	0.72	0.30	1.74	0.471	0.64	0.25	1.60	0.344
Irregular EMJ	8.15	3.75	17.72	<0.001^*∗*^	20.00	5.78	69.26	<0.001^*∗*^	25.47	6.74	96.25	<0.001^*∗*^
Focal adenomyomas	4.70	1.93	11.45	0.001^*∗*^	7.87	2.69	22.98	<0.001^*∗*^	7.69	2.54	23.28	<0.001^*∗*^

EMJ: endometrial-myometrial junction; OR: odd's ratio; LL: lower limit; UL: upper limit. ^*∗*^Statistically significant finding.

**Table 4:** Univariate analysis of the associations between demographic data, clinical symptoms, disease severity, and number of sonographic features of adenomyosis in the study group.

	Any adenomyosis feature				Five or above features			
	OR	95% CI for OR		*p*	OR	95% CI for OR		*p*
		LL	UL			LL	UL	
Age	1.14	1.01	1.29	0.031	1.04	0.97	1.11	0.291
BMI	1.18	0.95	1.46	0.123	1.03	0.94	1.13	0.520
Previous delivery	3.64	0.73	18.15	0.115	0.67	0.3	1.57	0.373
Previous cesarean	1.36	0.16	11.77	0.782	1.04	0.3	3.56	0.947
Dysmenorrhea	1.43	0.15	13.22	0.755	0.99	0.21	4.71	0.993
Dyspareunia	0.41	0.08	2.06	0.280	0.63	0.26	1.52	0.305
GI complaints	0.46	0.11	1.91	0.286	1.32	0.57	3.03	0.515
Urinary complaints	1.68	0.33	8.52	0.529	0.88	0.35	2.2	0.789
Infertility	1.21	0.28	5.21	0.800	**3.19**	**1.25**	**8.17**	^**∗**^0.015
ASRM score	1	0.98	1.02	0.873	**1.01**	**1**	**1.02**	^**∗**^0.046
ASRM stage	0.91	0.5	1.68	0.772	1.32	0.91	1.9	1.32

ASRM: American Society for Reproductive Medicine; BMI: body mass index; OR: odd's ratio; GI: gastrointestinal; OR: odd's ratio; LL: lower limit; UL: upper limit. ^*∗*^Statistically significant finding.
